# A Review of Chlamydial Infections in Wild Birds

**DOI:** 10.3390/pathogens10080948

**Published:** 2021-07-28

**Authors:** Helena S. Stokes, Mathew L. Berg, Andrew T. D. Bennett

**Affiliations:** Centre for Integrative Ecology, School of Life and Environmental Sciences, Deakin University, Geelong, VIC 3216, Australia; mathew.berg@deakin.edu.au (M.L.B.); andy.bennett@deakin.edu.au (A.T.D.B.)

**Keywords:** *Chlamydia*, *Chlamydia psittaci*, psittacosis, chlamydiosis, birds, *Chlamydiales*, bacteria, zoonoses

## Abstract

The *Chlamydia* are a globally distributed genus of bacteria that can infect and cause disease in a range of hosts. Birds are the primary host for multiple chlamydial species. The most well-known of these is *Chlamydia psittaci*, a zoonotic bacterium that has been identified in a range of wild and domesticated birds. Wild birds are often proposed as a reservoir of *Chlamydia psittaci* and potentially other chlamydial species. The aim of this review is to present the current knowledge of chlamydial infections in wild avian populations. We focus on *C. psittaci* but also consider other *Chlamydiaceae* and *Chlamydia*-related bacteria that have been identified in wild birds. We summarise the diversity, host range, and clinical signs of infection in wild birds and consider the potential implications of these infections for zoonotic transmission and avian conservation. Chlamydial bacteria have been found in more than 70 species of wild birds, with the greatest chlamydial diversity identified in Europe. The Corvidae and Accipitridae families are emerging as significant chlamydial hosts, in addition to established wild hosts such as the Columbidae. Clarifying the effects of these bacteria on avian host fitness and the zoonotic potential of emerging *Chlamydiales* will help us to understand the implications of these infections for avian and human health.

## 1. Introduction

The *Chlamydia* are a diverse genus of gram-negative, intracellular bacteria in the family *Chlamydiaceae* and order *Chlamydiales*, which share a unique biphasic development cycle of replication [1,2,3]. To date, 14 species have been proposed or formally classified, with four additional uncultured candidate species also proposed [4,5,6]. They have varying degrees of host specificity: some chlamydial species (‘chlamydial’ hereafter referring to any species within the order *Chlamydiales*) have only been reported in one host species, whereas others have been documented in multiple species of wild and domestic hosts, including humans [1].

One of the most well-documented zoonotic *Chlamydia* species, for which birds are the primary host, is *Chlamydia psittaci* [4,7]. *C. psittaci* is a zoonotic species that can infect and cause a severe disease in humans, termed psittacosis, which can result in pneumonia in up to 83% of cases and significant mortality if untreated [8,9]. *C. psittaci* is a globally distributed pathogen, to which more than 450 bird species from 30 different orders are known to be susceptible [10]. *C. psittaci* infection is particularly common in captive parrots and cockatoos (order Psittaciformes), where prevalence is between 16% and 81%, and in captive doves and pigeons (order Columbiformes), where prevalence is between 13% and 95% [7]. *C. psittaci* is also often found in poultry and is considered endemic in the turkey industry [11]. Additional orders of infected wild birds include the Lariformes (gulls) and Anseriformes (ducks and geese) [12]. Signs of disease in infected birds (termed psittacosis or avian chlamydiosis) can include lethargy, respiratory disease, anorexia, and conjunctivitis [13,14,15], although infection can also be subclinical [7]. *C. psittaci* strains tend to be host-specific [7], with pathogenicity dependent on host species as well as individual condition [14]. In humans, the pathogenicity of *C. psittaci* infection is also highly variable: symptoms can range from mild, nonspecific illness (such as chills and a headache) to severe systemic illness [16,17]. Subclinical infection is also common, and it is likely that many human cases remain unreported or are misdiagnosed [15]. Most reported cases of human psittacosis are thought to result from direct contact with wild or captive birds or bird material, such as through handling infected birds or inhalation of respiratory secretions or faecal particles [15].

Wild birds have long been proposed as a natural reservoir of *C. psittaci* infection [18]. However, direct evidence to support this hypothesis has been lacking. There has been relatively little targeted surveillance undertaken of wild bird populations, despite evidence supporting links between infections and mass mortality events [19] as well as zoonotic transmission to humans [9]. With the recent characterisation of several new avian *Chlamydia* species (namely *C. gallinacea*, *C. avium*, and *C. buteonis*) [4,5] as well as the proposition of novel transmission routes (specifically, transmission from wild birds to horses and then to humans) [20,21] the role of wild birds in chlamydial epidemiology warrants further discussion.

In this review, we present the current knowledge of chlamydial infections in wild birds worldwide. Our review addresses an important gap in the literature, as previous reviews of avian chlamydial infections have focused primarily on a captive bird or human disease perspective (e.g., [11,15]) or are confined to one geographic region or host species (e.g., [22]). We primarily discuss *C. psittaci*, as this is the most studied species; however, we also review the available data on other *Chlamydiaceae* and *Chlamydia*-related bacteria. We discuss the diversity, host range, and clinical signs of chlamydial infections in wild birds, and we consider the potential implications for zoonotic transmission and avian conservation.

## 2. Chlamydial Diversity in Wild Birds—The Known and the Novel

### 2.1. Chlamydia psittaci

*C. psittaci* is the best-studied avian *Chlamydia* species and has been isolated from several specimens across multiple host species (Section 3 ‘Host Range of Chlamydial Infections in Wild Birds’) and locations (Section 4 ‘Global Chlamydial Distribution’). *C. psittaci* is genetically variable, and genotypes have been designated based on outer membrane protein A (*ompA*) sequences. Genotypes A to F, E/B, M56, and WC were the first to be described [23,24,25], with further *ompA* genotypes proposed using newer sequencing techniques [26]. Genotypes A to F and E/B are primarily avian strains [24], whereas genotypes M56 and WC have been isolated from mammals [25]. Genotypes A and F are generally associated with psittacine birds, genotype B with pigeons, genotypes C and E/B with waterfowl, genotypes D and C with turkeys (*Meleagris gallopavo*), and genotype E has been isolated from a variety of bird species [7]. More recent genomic analysis has shown that genotype A strains belong to a lineage termed the 6BC clade, which is generally considered the most pathogenic [27,28]. While many of the avian *C. psittaci* genotypes were initially isolated from captive populations, several of these genotypes have also been found in wild birds [28,29,30]. Genotypes E and B are particularly common in feral pigeons (*Columbia livia domestica*) [30,31]. Genotype A has been isolated from a diverse range of wild hosts, including parrots (e.g., crimson rosellas (*Platycercus elegans*) and galahs (*Eolophus roseicapillus*)) [32,33], passerines (including robins (*Erithacus rubecula*), dunnocks (*Prunella modularis*) and great tits (*Parus major*)), raptors (including Eurasian sparrowhawks (*Accipiter nisus*) and common buzzards (*Buteo buteo*)) [34], and fulmars (*Fulmarus glacialis*) [30,35]. The more recently proposed genotype 1V appears to be associated with the Corvidae family [34,36]. Interestingly, genotype M56 (originally associated with mammals) has recently been found in association with wild raptors [34,37] in Switzerland and the USA.

### 2.2. Other Chlamydia and Chlamydia-Related Bacteria (CRB)

Since the identification of *C. psittaci*, several new *Chlamydia* species have been described in birds, and it is likely that some older reports of *C. psittaci* infections are actually infections with other *Chlamydia* species, particularly studies based only on serology [7]. Here we briefly describe the other chlamydial species that have been isolated from wild birds, with information available from captive birds for context where appropriate. *C. gallinacea* was first reported in domestic chickens (*Gallus gallus domesticus*) in 2009 [38] and was formally characterised in 2014 [39]. *C. gallinacea* has to date primarily been associated with poultry, having been detected in chickens and other poultry species in several countries globally [40,41,42,43,44], although it has been reported in one captive Passeriformes bird in Argentina [45]. Within the last four years, *C. gallinacea* has also been identified in wild birds, in two parrot species in Australia [33,46] and woodcock (*Scolopax rusticola*) in South Korea [36]. *C. avium* was also originally identified in captive birds, namely parrots and pigeons [39] with more recent isolation from wild Columbiformes [47,48] and a wild ring-necked parakeet (*Psittacula krameri*) [49]. Other recently described avian *Chlamydia* species include *C. buteonis*, isolated from a red-shouldered hawk (*Buteo lineatus*) [5], and a *Candidatus* species, *Ca.* C. ibidis, isolated from sacred ibises (*Threskiornis aethiopicus*) [50] and crested ibises (*Nipponia nippon*) [51]. Several chlamydial species primarily associated with human and other mammalian hosts have also been identified in wild birds, including *C. trachomatis* and *C. abortus* [29,52], as well as additional *Chlamydiaceae* which could not be defined to species level [29].

In addition to the *Chlamydiaceae*, there are several other related families within the *Chlamydiales* order, often described as ‘*Chlamydia*-related bacteria’ (CRB) [1]. There are eight additional families currently described. CRB are increasingly being identified in mammalian hosts [53,54,55] and have also been identified in birds, specifically poultry [56], and among wild birds, seabirds [57], and parrots [46]. Many of these emerging CRB are thought to cause disease in their respective hosts or humans [1,58], so the presence of these bacteria in wild hosts may be of zoonotic significance.

## 3. Host Range of Chlamydial Infections in Wild Birds

### 3.1. Parrots

The parrots and cockatoos (comprising the order Psittaciformes; hereafter referred to as parrots) consist of around 400 species [59], and in captivity they are frequently infected with *C. psittaci* [7]. The published studies of *Chlamydia* species in wild parrots are listed in Table 1. Most studies of wild parrots, as with other taxa, have focused on *C. psittaci* [7]. However, because some of the diagnostic methods described are not species-specific, they may have detected DNA from other chlamydial species or antibodies against other chlamydial species [33]. Apart from a few reports (e.g., [60,61]), chlamydial prevalence in wild parrots is usually below 10% (Table 1) and therefore much lower than the prevalence reported in captive parrots, which can be as high as 80% [7]. Reported prevalence estimates can vary depending on the sample type or diagnostic method used. For instance, in wild hyacinth macaw (*Anodorhynchus hyacinthinus*) nestlings, *C. psittaci* prevalence was 27% in cloacal swabs but only 9% in tracheal swabs in the same individuals [62]. In two other studies of wild parrots, a lower *C. psittaci* prevalence was identified from PCR analysis compared to sequencing [33,63].

Molecular analyses have demonstrated that the majority of *C. psittaci* strains identified in wild Australian parrots are in the 6BC clade [28,32,63]. Successful sequencing of *C. psittaci* strains has not, to our knowledge, been carried out for other wild parrot populations. The identification of the 6BC clade in wild Australian parrots suggests that wild hosts could be a reservoir of this clade, which is highly virulent in humans and has potential public health implications [28]. Other chlamydial species identified in wild parrots include *C. avium* in a single wild ring-necked parakeet in France [49] and *C. gallinacea* and other *Chlamydiales* (e.g., *Parachlamydiaceae*) in crimson rosellas and galahs in Australia [46,64].

### 3.2. Pigeons

The pigeons and doves (order Columbiformes) are a major host of *C. psittaci* infections, with genotype B considered to be endemic in this order [7,65]. Consequently, several populations of feral pigeons have been tested for *C. psittaci* (and more recently, other chlamydial species) worldwide [22,66,67]. A 2009 review of European studies reported evidence of *C. psittaci* infection in feral pigeons in 11 countries [22]. *C. psittaci* has since been identified in European feral pigeon populations in further studies [47,48]. Surveillance of feral pigeon populations for *C. psittaci* has also been carried out outside Europe, in countries including Brazil [68,69], Japan [67], and Thailand [70,71]. In Australia, *C. psittaci* has been isolated from an individual spotted dove (*Streptopelia chinensis*), and a strain primarily associated with Columbiformes was also isolated from infected equine samples [72]. The majority of *C. psittaci* strains identified in wild pigeons and doves across Europe are in genotypes B and E [30,31,47], and genotype B has also been identified in pigeons in Thailand [71]. *C. avium* has now also been identified in several feral pigeon populations in Europe [39,47,48] at prevalences ranging from 0.9% to 36.6% [47,48], with one study in the Netherlands detecting *C. avium* at a higher prevalence than *C. psittaci* [48]. Other chlamydial species have also been identified in feral pigeon populations, such as *C. pecorum* in Japan [67] and *C. pecorum*, *C. abortus*, and *C. trachomatis* in Germany [66]. While the majority of studies only report *C. psittaci* and *C. avium* infections, this may simply reflect testing protocols, as pigeons have been tested for these chlamydial species most frequently (Table 1).

### 3.3. Other Wild Bird Species

*Chlamydiaceae* have been found in a wide variety of other wild bird species and appear to be fairly prevalent in birds in the Anatidae (duck) family (19.7–58.0%), where *Chlamydiaceae* have been identified in at least five different species [12,29,73] and the Corvidae (crow) family (13.4–23.7%) where *Chlamydiaceae* have been isolated from at least six species [29,34,36]. Many seabirds are also infected, with *Chlamydiaceae* detected in at least seven different species from three different families, including the Laridae (gulls; prevalence of up to 13.6% in European herring gull (*Larus argentatus*)), Sulidae (gannets and boobies; up to 41.3% in the Northern gannet (*Morus bassanus*) [12]) and the Procellariidae (fulmars; where prevalence is up to 21% according to location [74]). Both the Anatidae and Corvidae families can have *C. psittaci* infections [12,29], and several seabird species, including fulmars, black-headed gulls (*Chroicocephalus ridibundus*), and Northern gannets, have been found infected with *C. psittaci* and *C. psittaci* related strains [12,57,74]. The Anatidae, Corvidae, and Laridae have also been reported with *C. abortus* and non-classified *Chlamydia* infections [29,34], and the Gruiformes (specifically, Eurasian coots (*Fulica atra*)) have tested positive for *C. trachomatis* [52]). Raptors within the Accipitridae family are increasingly being tested for chlamydial bacteria and have tested positive for *C. psittaci*, the novel *C. buteonis*, and for novel CRB [5,75,76]. Further host species are increasingly being found with other *Chlamydia* species, such as woodcock with *C. gallinacea* [36]. Gulls have been found harbouring novel *Chlamydiales* outside the *Chlamydia* genus [57,77]. Recently, wild greater flamingos (*Phoenicopterus roseus*) in France have been found harbouring two newly proposed *Chlamydiaceae* species within a newly proposed genus, *Chlamydiifrater* gen. nov. [78]. It is likely that a wide variety of wild bird species are carrying other known and novel *Chlamydiales* [79], which may become evident with increased testing and molecular analyses.

Estimates of chlamydial prevalence have varied greatly between studies, even within the same host lineages (family or order). For example, across the Passeriformes, *Chlamydiaceae* prevalence was only 0–0.4% in some European surveillance studies [30,31]. In contrast, chlamydial prevalence has also been reported at 23% (5/22 positive) in the Passeriformes [52] and prevalence as high as 54% reported within the Paridae family [80]. Additionally, a retrospective study in the U.K. found several passerine birds (including dunnocks, great tits, and blue tits (*Cyanistes caeruleus*)), which tested positive for *C. psittaci* [81]. There are several reasons why infection levels may fluctuate within a family, species, or population; seasonal variation in prevalence or seroprevalence has been found in gannets and crimson rosellas [12,46], with inter-annual variation reported in pigeons [66]. A limitation of studies to date that have tested wild species other than Columbiformes and Psittaciformes is that they are largely opportunistic, for instance, carried out on veterinary submissions or hunted birds (e.g., [32,52]) or as part of a sampling program for other diseases (e.g., [30]). This often results in a large total number of individuals being tested, but often only a small number of each species, limiting the scope of intra-species or other intra-taxa comparisons of prevalence or chlamydial diversity.

### 3.4. Studies Where Chlamydia Have Not Been Found

While *C. psittaci* and other chlamydial species have been detected across a wide host range, there are host species and studies in which very few or no birds have tested positive (Table 1). In two of the larger surveillance studies carried out in the last five years (in Switzerland and Australia), less than 1% of wild birds tested positive for *C. psittaci* or other *Chlamydia* [31,32]. Each study tested more than 40 different species from more than 20 families, a combined total of more than 600 individuals [31,32].

Early psittacosis outbreaks in Europe and the USA were attributed to the import of wild South American parrots [7]. However, of five studies of wild South American parrots, only two found evidence of *C. psittaci* infection ([62,82]; Table 1). While some of these South American studies were of nestlings [62,82,83] that may have limited exposure to *C. psittaci*, it is interesting that neither of the two studies of adults found any positive individuals [84,85].

There are many potential reasons for why some host species or populations are less likely to suffer chlamydial infections. These include host species variation in susceptibility to infection and disease, as well as seasonal or inter-annual variation and geographic variation in infection rates, as described above [12,66,69]. Alternatively, *C. psittaci* and other *Chlamydia* may not be detected in wild birds if a host species or population suffers severe acute disease, resulting in rapid death and so making detection unlikely [86].

## 4. Global Chlamydial Distribution

### 4.1. Europe

There have been studies of chlamydial presence and diversity in wild birds in several countries in Europe, with multispecies surveillance carried out to a larger degree in Switzerland and Poland (Table 1). Many reports from Europe are of feral pigeon populations, but there has also been testing of other avian taxa, including waterfowl [29], songbirds [81], corvids [29,34], raptors [34,93,94], seabirds [12,74], feral ibis [50] and ring-necked parakeets [49]. Indeed birds have been found positive for *Chlamydiales* in all these taxa, with a range of different chlamydial organisms identified. *C. psittaci* has also been identified in most of the taxa above. *C. avium* has been found in pigeons in Switzerland, Italy, and the Netherlands [47,48,97]. *C. avium* has also been isolated from a ring-necked parakeet in France [49] and from a single mallard (*Anas platyrhynchos*) in Poland [29]. *C. gallinacea*, although widespread in poultry across Europe [38,40,98], has not yet been identified in European wild bird populations, and neither has *C. buteonis*, a recently described species, although screening for *C. buteonis* has now been undertaken in Switzerland [34]. *Ca. C. ibidis*, the main other chlamydial species affecting birds, was first isolated from wild birds in Europe (specifically, feral sacred ibises [50]). Non-classified *Chlamydiaceae* have been found in waterfowl and corvids in Poland [29].

### 4.2. Asia

Across Asia, feral pigeons have been tested for *C. psittaci* in Thailand, India, Japan, Korea, and Iran, with prevalence ranging from 1% to 25% [36,61,70,71,90,99]. Other species testing positive for *C. psittaci* include ring-necked parakeets (26.3%) and crows (18%) (*Corvus splendens*) in India [61], house sparrows (*Passer domesticus*) (14.8%) in Iran [90], and rooks (*Corvus frugilegus*) and Korean magpies (*Pica serica*) in South Korea [36]. There is little evidence of large numbers of any other avian species being tested. Other chlamydial species have been identified in wild birds in Asia, including *C. pecorum* and *C. gallinacea* (in pigeons and woodcock, respectively) [36,67] and an uncharacterised chlamydial species closely related to *C. avium* (in pigeons) [71]. Interestingly, while there are numerous studies of *Chlamydia* in captive birds in China [42,100,101,102], there is little evidence of testing wild birds. Since a diverse range of *Chlamydia* has been identified in Chinese poultry [42], it is plausible that a diverse range of *Chlamydiales* both within and outside the *Chlamydia* genus are circulating in wild birds in China and in other countries across Asia.

### 4.3. North America

In the USA, there have been suspected epizootics of psittacosis, in juvenile white-winged doves (*Zenaida asiatica*) in Texas [103], and in California gulls (*Larus californicus*) and ring-billed gulls (*Larus delawarensis*) in North Dakota [19], with *C. psittaci* found in many of the birds sampled at necropsy [19,103]. In recent years, a mortality event in rosy-faced lovebirds (*Agapornis roseicollis*) prompted screening of wild birds at feeders in Arizona, where several bird species (including feral pigeons, house sparrows, and Inca doves (*Columbina inca*)) tested positive for *C. psittaci* [88]. There has been some raptor surveillance in the USA; a *Chlamydiaceae* prevalence of 1.4% was found in wild hawks in the *Buteo* genus [37], with the chlamydial species identified later characterised as *C. buteonis* [5]. Additionally, *C. psittaci* and another member of the *Chlamydiales, Candidatus* Rhabdochlamydia spp., were identified in an osprey (*Pandion haliactus*) and a red-tailed hawk (*Buteo jamiacensis*), respectively [75]. In Canada, feral pigeons have been found infected with *C. psittaci* [104]. To our knowledge, the other avian *Chlamydia* (*C. gallinacea* and *C. avium*) have not been found in wild birds in North America: given that *C. gallinacea* has been identified in free-ranging poultry in the USA [44], it is plausible that the absence of other avian *Chlamydia* in wild birds reflects a lack of testing, rather than true absence.

### 4.4. South America

In South America, at least five wild parrot species have been tested for chlamydial infection, with these species possibly targeted due to the high prevalence of *C. psittaci* in many captive parrots [7] and historical cases of human psittacosis being linked to the importation of South American parrots [15,105]. Wild red-tailed Amazon parrot (*Amazona brasiliensis*), blue-fronted Amazon parrot (*Amazona aestiva*), and hyacinth macaw nestlings have been tested in Brazil [62,82,83]. Prevalence was 0–1.2% in red-tailed Amazon parrot nestlings [82,83] compared to 6.3% and 26.7% prevalence in blue-fronted Amazon nestlings and hyacinth macaws, respectively [62]. There are few studies where wild adults have been sampled (Table 1). All adult studies included only serological testing; there was no evidence of chlamydial antibodies found in dusky-headed parakeets (*Aratinga weddellii*) or tui parakeets (*Brotogeris sanctithomae*) in Peru [85] or blue-fronted Amazon parrots in Bolivia [84]. There is thus little to no evidence of chlamydial infection in wild adult South American parrots, although *C. psittaci* has been identified in captive populations of adult Amazon parrots [106], including in birds recovered from the wildlife trade, where at the same location, *C. psittaci* caused up to 97% mortality in nestlings [107].

In Brazil, studies have tested feral pigeons for *C. psittaci*, with prevalence ranging between 11.7% and 16.8% [68,69,108]. There is substantial variation between study locations; in São Paulo prevalence of *C. psittaci* was 37.8% compared to only 6.1% in Botucatu [69]. Although there are several Columbiformes species present across South America, including introduced feral pigeons, there are few studies from other countries testing feral pigeons or other Columbiformes for *C. psittaci*. Interestingly, 5.9% (6/102) of Galapagos doves (*Zenaida galapageoensis*) tested positive for *C. psittaci* on the Galapagos Islands, Ecuador, although none of the 28 feral pigeons tested positive in the same study [92]. Considering birds outside the Psittaciformes and Columbiformes, *C. psittaci* has been identified in seabirds in Chile during screening carried out as a comparison with surveillance in Antarctica [77]. There is little to no evidence of testing for chlamydial species other than *C. psittaci* in South America or molecular sequencing of any chlamydial strains identified.

### 4.5. Australasia/Oceania

Signs of psittacosis were reported in wild Australian parrots obtained from dealers as early as the 1930s [109] and again in wild parrots during the 1950s, at 10.6% prevalence, with prevalence varying between host species [60]. More recent estimates of *C. psittaci* prevalence in wild Australian parrots are often lower, usually ranging between 0% and 1.8% [32,63,91], although some studies have reported prevalence estimates between 6.2% and 9.8% in common species such as galahs and crimson rosellas [33,46]. While the majority of wild bird studies to date in Australia have focused on parrots (Table 1), waterfowl, and other host species have been sampled [32,91]. Where species other than parrots have been tested, no birds tested positive in either study except for a single superb lyrebird (*Menura novaehollandiae*) [32,91]. In New Zealand, *C. psittaci* has been identified in feral pigeons and other Columbiformes, in a native hihi (*Notiomyces cincta*) [89], and in two wild duck species sampled in a wildlife rehabilitation centre [110]. As in most other regions, the majority of Australasian studies have only tested for *C. psittaci*, without testing for other *Chlamydiales*. However, two recent studies in Australia have reported a greater diversity of chlamydial organisms, including *C. gallinacea* in two wild parrot species [46,64], as well as *Chlamydiales* from other orders including the *Parachlamydiaceae* [33,46]. As next-generation sequencing (NGS) is increasingly used to test samples from wild Australian birds [28,63], it is likely that a greater diversity of *Chlamydiales* may soon be described.

### 4.6. Africa and Antarctica

There are very limited data available on the distribution of chlamydial bacteria in wild birds in Africa. Surveillance of pelicans (*Pelecanus onocrotalus*) in South Africa found no positive individuals [87]. There is limited evidence to suggest that *Chlamydia* are present in wild birds in Egypt [111]. However, we were unable to find any other studies of chlamydial infection in wild African birds. Interestingly, chlamydial organisms have been identified in chinstrap penguins (*Pygoscelis antarcticus*) and seabirds from Antarctica [77] with an 18% prevalence of order *Chlamydiales*, although *C. psittaci* was not identified.

## 5. Host Disease and Fitness

### 5.1. Signs of Disease and Survival

The impacts of *C. psittaci* infections in wild birds are rarely documented [79], with reported effects of other chlamydial species even rarer. Many wild bird populations are thought to harbour chlamydial infections without being visibly affected [7]. However, suspected epizootics have occurred, such as in white-winged doves and various gull species in the USA [19,103], suggesting that chlamydial disease can also impact wild populations [112]. For the majority of studies discussed in this review, signs of infection were not recorded (Table 1), and across the literature, the majority of reported clinical signs are from captive birds (principally poultry and parrots) [11,14]. However, there are some reports of clinical signs in wild individuals, primarily from birds tested at rehabilitation centres or wildlife rescue clinics [95,107]. Wildlife health centres in the U.K. and Australia have reported wild *C. psittaci*-positive birds (specifically pigeons, a crimson rosella parrot and a superb lyrebird) being ‘emaciated’ [32,95], with the crimson rosella also presenting with diarrhoea [32]. Surprisingly, neither of these studies reported overt signs of respiratory distress in infected birds, although these are among the main disease signs found in captive birds [13,14]. Infected parrots in other Australian studies have also been found emaciated with diarrhoea [28,113,114]. However, some of these reports [28,114] are case studies of individuals who presented with severe clinical signs and were subsequently specifically tested at veterinary clinics. As such, they are likely to be cases with a very high bacterial load, which may not be representative of naturally infected wild individuals. Contrasting to this, in recent studies of apparently healthy wild Australian parrots, there was no significant association found between infection and three indices of body condition across the four host species tested [33,46]. There are few observations of infected wild parrots elsewhere globally for comparison, but hyacinth macaw nestlings in Brazil, which had between 9% and 27% prevalence depending on sample type, also showed no clinical signs [62]. However, captive studies have demonstrated that the same host species can suffer both mild and severe consequences of chlamydial infection. For example, high rates of *C. psittaci* infection have been found in healthy captive Amazon parrots (*Amazona* genus) in breeder collections [106] but the same genus has also suffered mortality rates of up to 97% when infected under stressful conditions [107]. Indeed, in most cases where *C. psittaci* has caused acute death or severe disease, for parrots at least, affected individuals were already stressed or immunocompromised, with many having recently been captured from the wild [107,115] or being co-infected with other pathogens, such as beak and feather disease virus (BFDV) [32].

It is generally considered that wild pigeons are relatively unaffected by *C. psittaci*, because they excrete *C. psittaci* without showing signs of disease [7,66]; moreover, pigeons in captivity often harbour subclinical infections with periodic shedding [18,22]. Some feral pigeon populations have only been sampled indirectly through faecal sampling, which limits understanding of the links between individual infection and disease signs [48,67]. Where there has been direct sampling (i.e., through using cloacal or choanal swab sampling [47,71]), some studies specifically state that there were no signs of infection ([68]; studies described in [22]). In most reports, however, disease signs were not recorded, and the effects of chlamydial infection on feral pigeon populations have not, to our knowledge, been systematically investigated. It is plausible that signs of disease in feral pigeons are similar to in captive pigeons, which often show clinical signs only when concurrent infections are present [116].

In the rare instances where clinical signs have been reported for other orders or species (i.e., other than Psittaciformes and Columbiformes) findings vary widely between studies and study populations. For example, a retrospective study in the U.K. carried out post-mortem examination of Passeriformes with signs of chlamydiosis and found evidence of histological lesions in 42% (8/19) birds that were *C. psittaci*-positive [81], suggesting that *C. psittaci* may also cause disease in passerine species including great tits and dunnocks. On the other hand, Holzinger-Umlauf et al. (1997) reported a high prevalence in tits (Paridae; 54%) and great tits specifically (53%) with no clinical signs [80], and Krawiec et al. (2015) similarly identified *C. psittaci*-positive passerine birds without signs of chlamydial disease [52]. Several factors may cause variability in clinical signs between populations. Individual conditions can cause marked variation in the course of infection, as shown by captive studies [14], and host species can suffer different disease signs when infected with different strains. An additional consideration is that the authors who carried out the retrospective post-mortem analysis only targeted passerine birds with clinical signs of disease; they did not test any clinically healthy birds [81]. Considering post-mortem studies from other species, there have been variable findings at necropsy; organ inflammation and hepatitis have been reported in pigeons and gulls [19,104], whereas other studies reported no pathological changes [52].

Concurrent infections of *C. psittaci* with other pathogens have been observed repeatedly in wild birds. More than half of the *C. psittaci*-positive passerine birds examined by Beckmann et al. (2014) had concurrent infectious diseases, including avian pox and trichomonas [81]. In other hosts, *C. psittaci* has also been found occurring concurrently with other infections, such as with BFDV in wild Australian parrots [32,63] and pigeon circovirus in feral pigeons [96]. There is a lack of longitudinal data available from wild birds to test whether chlamydial infection may predispose individuals to other infections (i.e., whether birds are infected with *C. psittaci* first) or whether birds immunosuppressed by other infections are then more likely to become infected with *Chlamydia*. Indeed, any repeated capture and testing of wild birds for their chlamydial infection status is rare. However, repeated testing has been carried out on recaptured tit species (*Parus* genus; Passeriformes) in Germany [80] and recaptured parrots (cockatoos and rosella (*Platycercus*) species) in Australia [33]. In both studies, recaptured individuals frequently changed in their *Chlamydia* status, suggesting that wild birds may suffer persistent infections and shed *Chlamydia* intermittently or may suffer repeated infections [33,80]. Persistent infections have also been proposed in Canada geese (*Branta canadensis*), which were found to have a high antibody prevalence (93.8%) in conjunction with lower levels of bacterial shedding, and no clinical signs [73].

The limited data available on chlamydial species other than *C. psittaci* (i.e., *C. gallinacea*, *C. avium*, or *C. buteonis*) in wild birds means that their effects on host health and survival are unknown. Indeed, the effects of these infections remain largely unknown even in captive birds [4], although chickens experimentally infected with *C. gallinacea* showed reduced bodyweight gain [42], and *C. avium* is hypothesised to cause depression, respiratory disease, and subsequent mortality in parrots [117].

### 5.2. Reproduction and Fitness

There is little information on whether chlamydial infections affect reproductive success in wild birds. One study of crimson rosellas found that breeding birds were much less likely to be infected compared to non-breeding birds [46], which suggests that infection may reduce the likelihood of breeding in this species. However, further data quantifying the effects of chlamydial infection on this population are needed to test this hypothesis; it is possible that the different infection rates were instead due to seasonal effects such as changes in social behaviour or breeding stress. Alternatively, there could be sub-lethal population-level effects of chlamydial infections, in addition to the reported epizootics [19,103]. Sub-lethal population impacts of infection are much less likely to be detected; wildlife disease sampling is generally biased towards mass die-offs [118]; hence, more subtle population-level effects are likely to be missed, as are sporadic cases with low-level mortality [13]. For instance, captive studies have shown that younger birds are generally more susceptible to *C. psittaci* infection than adults [14], with evidence to suggest that the same could be true in some wild bird populations [46,103]. A chlamydial epizootic affecting wild white-winged doves resulted in an apparent population reduction of approximately 75%, where juvenile white-winged doves were disproportionately affected [103]. Such events could affect recruitment and breeding in subsequent years and may result in an altered population age structure. However, apart from the two reports described above, we were unable to find any observations or discussion of whether chlamydial infections may affect reproduction and population size.

## 6. Conservation Implications

Since *C. psittaci* is known to cause severe disease in at least some avian hosts, it is plausible that it may result in population declines in wild bird populations, particularly when populations comprise highly susceptible hosts (such as immunocompromised or inbred individuals) or when combined with additional stressors or concurrent infections. The same may be true for other *Chlamydiales* species, although their pathogenicity remains to be investigated. Chlamydial infection in certain avian populations may therefore be of conservation concern, particularly in hosts such as parrots, which are one of the most highly threatened bird orders [59], are known to suffer frequent *C. psittaci* infections [7,33] and can subsequently suffer high mortality [107,115]. In parrots and other birds, small, highly threatened populations that may have lost endemic pathogens may be particularly at risk of infection via pathogen spill-over from sympatric species, as suggested recently with BFDV in the critically endangered orange-bellied parrot (*Neophema chrysogaster*) [119].

Conservation concerns may also arise from the host specificity in chlamydial strains, with some genotypes occurring more frequently in certain orders of birds, and varying in virulence [7]. Strains that cause no clinical signs in one bird species can potentially cause severe disease in other host species; for example, in captive turkeys in the USA, experimental inoculation with a turkey strain from the same region caused much less severe disease compared to a parrot strain, and even compared to a turkey strain from Europe [120]. Consequently, if chlamydial strains associated with particular hosts are introduced into naïve populations or alternative hosts, they could potentially cause severe disease and population declines. Invasive species and infectious diseases are well-known causes of species declines and extinctions [121], and if an invasive species has a high chlamydial infection rate, it is possible they could infect a naïve host population and cause severe population impacts. For example, feral Canada geese are a widespread invasive species in Europe [122], which have a *C. psittaci* prevalence of up to 58%, seroprevalence of 94%, and show no clinical signs [73]. Species such as this may thus represent a potential reservoir for other host species. A newly proposed chlamydial species, *Ca.* C. ibidis has been isolated from another highly invasive species, the sacred ibis [50]; it is plausible that novel chlamydial species from this host could be transmitted to naïve hosts.

In addition, human-induced habitat change can increase stress on wild populations [123]. When the availability of suitable habitat for birds is reduced (such as through urbanisation, agricultural development, or climate change), birds can be forced into closer proximity with conspecifics and other species, resulting in higher population densities and increased social interaction, as well as changes in food availability and nutritional deficiencies [123]. Increased stress can affect host condition and reduce tolerance of infection [124] and can make birds more susceptible to chlamydial disease [15]. Furthermore, as stress increases chlamydial shedding [14,15], stressed birds may shed *Chlamydia* more frequently, favouring further transmission and environmental contamination. Habitat destruction and other stressors (including human encroachment) are already suspected to exacerbate chlamydial infection and disease in koalas (*Phascolarctos cinerus*), which are otherwise often asymptomatic, with little impact of chlamydial infection on wild populations [124,125]. The presence of additional stressors may similarly exacerbate impacts of chlamydial infection in wild bird populations.

## 7. The Role of Wild Birds in Zoonotic Transmission

### 7.1. Evidence and Risk Factors for Zoonotic Transmission

Most reported cases of human psittacosis result from direct contact with birds or bird material, such as through handling infected birds or inhalation of respiratory secretions or faecal particles [15]. Most cases of human psittacosis are suspected to be contracted through contact with captive birds [126,127,128]; however, there are suspected cases due to direct and indirect contact with wild birds; a systematic review of human psittacosis case-control studies revealed that 16% of the articles included considered that direct or indirect contact with wild birds was a potential source of infection [129]. There are several cases where human infection is suspected from environmental exposure to, or handling of, feral pigeons [22,130,131,132,133], although direct testing of feral pigeon populations within the same locality is rarely carried out to test this hypothesis.

Environmental exposure or direct contact with wild birds other than feral pigeons have been hypothesised sources of infection following case-control studies in Sweden and Australia. In Sweden, two studies identified cleaning wild bird feeders and exposure to bird faecal material to be risk factors for human psittacosis [134,135]. A few years prior to this, bird ringers in Sweden were tested for chlamydial antibodies; however, none showed evidence of seroconversion to *C. psittaci*, despite a history of directly handling birds [136]. In Australia, direct contact with wild birds (primarily parrots) and mowing lawns without a grass catcher were identified as risk factors for human infection [8,9,137]. In the Blue Mountains, a region of Australia where human psittacosis is considered endemic, the same strain of *C. psittaci* was identified in six humans and a wild crimson rosella parrot [28], and three humans working in a veterinary clinic contracted psittacosis following their handling of an infected parrot [114]. Studies from this region of Australia therefore provide some of the best evidence for possible zoonotic transmission of *C. psittaci* from wild birds to humans.

While most human cases of psittacosis with a confirmed association with wild birds have involved close contact with and handling of wild birds (e.g., wild birds brought into vet surgeries [109]), as discussed above, some cases have been linked to cleaning wild bird feeders [135] and mowing lawns without a grass catcher [9]. This highlights the potential benefits of greater public awareness and perhaps proactive human or wild bird surveillance in communities suspected at risk (such as the proactive surveillance described in [8]). This surveillance could target locations where humans come into close contact with birds through feeding, such as designated wildlife feeding areas or recreational sites where birds and humans congregate in close proximity [138,139]. Urbanisation can also increase the frequency of human-wildlife interactions [140]. In many countries, known chlamydial hosts such as feral pigeons and ring-necked parakeets thrive in city environments [141,142], and in Australia, the abundance of some native parrot species (such as rainbow lorikeets (*Trichoglossus moluccanus*) and sulfur-crested cockatoos (*Cacatua galerita*)) are also increasing in suburban areas [143,144]. At such locations where habitat change or urbanisation results in an increased human presence and shifts in local bird abundance, there may be increased opportunities for zoonotic disease transmission, which may warrant targeted surveillance. An additional factor to consider when assessing zoonotic transmission risk is seasonality; only a few studies of wild birds investigate how chlamydial prevalence changes across seasons and years (Table 1; [10,43,63]), although temporal variation in host infection rates could affect the likelihood of zoonotic spill-over.

### 7.2. Transmission Involving Poultry and Agriculture

It is plausible that wild birds could be a reservoir for chlamydial infections in poultry, and subsequently of health concern to farm workers and consumers. Wild birds have previously been implicated in poultry infections [98,145], with transmission proposed via environmental contamination of feed or equipment [15]. On the other hand, serological analysis indicates that chlamydial exposure can be very high in poultry (90%–100%) [146], and infection is considered endemic in some poultry systems, such as in the turkey industry [11]. It is possible that poultry species are able to maintain chlamydial infection within a flock without the need for any maintenance reservoir host. Indeed, pathogen surveillance in sympatric wild and domesticated felid species in the USA suggests that inter-species transmission is relatively rare and that following sporadic cross-species transmission events, pathogen transmission becomes self-sustaining within the recipient host population [147]. Given that direct transmission is likely to be the most common route of chlamydial transmission and poultry are kept at relatively high densities (particularly at night, when kept in sheds), self-sustaining transmission is likely to facilitate rapid transmission and maintenance of chlamydial infection in poultry systems. Finally, while there is usually a focus on wildlife species being the reservoir host, it is also plausible that chlamydial bacteria are transmitted from poultry to wild birds, particularly on free-range farms where feeding stations are accessible to wild birds. For instance, *C. gallinacea*, a chlamydial species primarily associated with chickens, has been identified in two wild Australian parrot species [46,64].

There is evidence for *C. psittaci* spill-over from wild birds to horses and thereby to humans in Australia, with a highly virulent strain of *C. psittaci* associated with equine reproductive loss [148] and subsequently disease in humans [21]. This study provided the first evidence for mammal to mammal transmission of *C. psittaci* [149] and has since prompted further testing of horses in Australia [150,151].

## 8. Recommendations for Future Research

It is well established that *C. psittaci* is present in wild bird populations, is globally distributed with a broad host range, and that other chlamydial bacteria are likely to be similarly widespread. However, the true prevalence of chlamydial infections in wild bird populations is often unknown, particularly outside Europe, where less chlamydial surveillance has been carried out. Globally, there is a need for more proactive screening or active surveillance of wild bird populations, as opposed to convenience sampling through veterinary or community submissions, wildlife rehabilitation centres, or faecal sampling. Such methods can be biased and may only detect a subsample of wild bird populations. There are some avian taxa, specific examples including waterfowl, crows, and raptors [29,34,73], which are quite frequently found positive for chlamydial infection but have received less attention as chlamydial hosts compared to parrots and pigeons. It may be worth screening more of these birds from different regions to test whether this is a global occurrence. This surveillance may be of particular importance given the identification of new chlamydial species (e.g., *C. buteonis*) [5] of unknown pathogenicity. Additionally, while wild birds are increasingly being tested for multiple chlamydial species (examples including [28,30,49]), some studies still only carry out targeted screening for *C. psittaci*. To thoroughly investigate the diversity of *Chlamydiales* present within a host, it would be useful to use a broad spectrum pan-*Chlamydiales* PCR and a combination of either species-specific PCR protocols or sequencing. Such an approach has been used to successfully investigate the diversity of *Chlamydiales* present in wild ungulate populations [55].

Longitudinal studies are needed to investigate the potential impact of *C. psittaci* and other chlamydial infections on bird survival, reproduction, and so fitness. Although the non-specificity and variability of clinical signs make investigating the impacts of chlamydial infection challenging, it would be useful to test for an association of strains with clinical signs in hosts of particular concern (for either zoonotic or conservation reasons) by recording clinical observations, taking relevant erythrocyte measures to assess haematological changes (for example, haematocrit) [14,152] and measuring changes in enzyme profiles and blood biochemistry [14]. These measures would be of greater value if individuals could be sampled more than once, in order to investigate pathological changes within the same individuals, and mark-recapture data could be used concurrently to assess survival [153]. Ideally, other pathogens would be screened for simultaneously to evaluate the impact of co-infections, particularly as evidence from several bird species indicates that chlamydial infections cause more severe disease in birds suffering concurrent infections [32,81,96]. It would be useful to measure indicators of breeding success and population health alongside this work, to evaluate the population-level impacts of infections. While such proposed investigations (particularly those involving recaptures) are logistically challenging in wild birds, they would provide useful insights into both individual and population effects of chlamydial infection, and such investigations would help to investigate any potential impact chlamydial infections may have on avian conservation.

Finally, in order to investigate suspected transmission pathways, including potential cases of zoonotic transmission, further phylogenetic comparisons are required, particularly of strains within and between hosts. NGS has greatly advanced the opportunities in this field. NGS techniques have already been employed to investigate potential sources of psittacosis outbreaks from wild birds in Australia [28,148] and were recently used to retrospectively investigate the *C. psittaci* strains present in fulmars following a human psittacosis epidemic that occurred several decades ago [35]. When zoonotic transmission is expected, ideally there should be a coordinated effort between health professionals, wildlife ecologists and veterinarians in order to carry out sampling of humans and wild birds within the same region and within a short time frame, with subsequent sequencing and phylogenetic analysis of any chlamydial strains identified.

## 9. Conclusions

Chlamydial bacteria have been found on every continent and have been isolated from at least 70 different species of wild bird. While the Psittaciformes and Columbiformes have long been known to harbour chlamydial infections, recent evidence suggests that families including the Corvidae and Accipitridae can also have a high prevalence, with a degree of host specificity in strains. Most chlamydial surveillance has been undertaken in Europe, which is reflected in the greater diversity of chlamydial organisms identified there; with increased surveillance carried out in other regions, it is likely that more chlamydial organisms will be identified and in a broader range of hosts. Most research has focused on zoonotic *C. psittaci*, but infections with *Chlamydiales* other than *C. psittaci* are proving to be more common than previously anticipated and sometimes more prevalent than *C. psittaci*. Increased understanding of the diversity and effects of these bacteria would be beneficial, including their zoonotic potential. Furthermore, although *C. psittaci* can cause disease in wild birds, there is scarce data on the effects of chlamydial infections on host fitness. It is possible that the majority of chlamydial infections in wild populations are commensal, at least for host-adapted strains, without negatively impacting either the host or sympatric species. However, the occurrence of epizootics, although rare, and the potential for severe disease suggests that chlamydial infections may also be relevant to avian conservation. As the world continues to be impacted by habitat destruction and environmental change, there is an urgent need to better understand fundamental disease ecology in wildlife hosts, particularly of pathogens that are known to be highly capable of host switching. Future surveillance of chlamydial infections in wild birds, such as the investigations outlined above, should offer benefits for wild birds, captive birds, and human health alike.

## Figures and Tables

**Table 1:** Studies of wild birds summarized in this review. Where a study included both captive and wild birds, we only report prevalence in wild birds. We have excluded case studies involving a single individual bird.

Host Species	Family	Location	Sample Source	Sample Size	Detection Method(s)	*Chlamydiales* Species Tested For	Key Findings	Disease Signs Reported? (Y/N/NR) **	Reference
Seabirds; 13 species, 4 orders *	Anatidae, Alcidae, Laridae, Procellaridae, Sulidae	France	Rehabilitation centre	195	PCR (cloacal swabs) Sequencing Multilocus sequence typing (MLST)	*C. psittaci* *Chlamydiaceae*	• 18.5% *Chlamydiaceae* prevalence (prev.) • Prev. varied between host spp.; Northern gannets *Morus bassanus*) had higher prev. compared to European herring gulls (*Larus argentatus*) and common murres (*Uria aalge*) • Seasonal variation in prev. (in Northern gannets) • *C. psittaci* identified in Northern gannets and herring gulls • Unclassified *Chlamydiaceae* also identified	N	Aaziz et al., 2015 [12]
48 species from 11 orders *	Psittacidae, Cacatuidae, Podargidae (…) *	Australia	Rehabilitation centre	229	PCR (live birds: choanal/cloacal swabs; dead birds: swabs from trachea and intestine/caecum) MLST	*C. psittaci*	• 1 crimson rosella (*Platycercus elegans*) and 1 superb lyrebird (*Menura novaehollandiae*) tested positive (0.7%) • All other wild birds tested negative	Y	Amery-Gale et al. 2020 [32]
Great white pelicans (*Pelecanus* *onocrotalus*)	Pelecanidae	South Africa	Live trapping	50	PCR (tracheal and cloacal swabs)	*C. psittaci*	• 0% *C. psittaci* prevalence	N/A	Assunção et al., 2007 [87]
Songbirds (Passeriformes); Pigeons and doves (Columbiformes) *	Paridae, Prunellidae, Turdidae (…) *	U.K.	Necropsy (selected based on clinical signs)	40	PCR (liver and spleen) Histology Immunohistochemistry	*C. psittaci*	• 53% *C. psittaci* prevalence • Nonspecific clinical signs observed (lethargy, fluffed up plumage) and emaciation • Concurrent disease in >50% cases • Genotype A present in all passerine cases	Y	Beckmann et al., 2014 [81]
16 species from 5 orders *	Cacatuidae, Psittacidae, Columbidae (…) *	Australia	Trapping and community submissions	278	Isolation (from liver) and inoculation Serology (Complement Fixation Test (CFT))	*C. psittaci* (methods not spp. specific)	• 10.6% prevalence (Psittaciformes) • 0.7% prevalence (all other species; 1 house sparrow (*Passer domesticus*) tested positive)	NR	Beech and Miles 1953 [60]
Peregrine falcons (*Falco peregrinus*) and white-tailed sea eagles (WTSE) (*Haliaeetus albicilla*)	Falconidae, Accipitridae	Sweden	Nestlings (breeding monitoring), adults (museum submissions)	319 (299 nestlings; 108 falcons and 191 WTSE, and 20 WTSE adults)	PCR (cloacal swabs) *ompA* sequencing	*C. psittaci* *Chlamydia*	• 1.3% *C. psittaci* prev. (*n* = 2 falcons, *n* = 2 eagles) • New strains of *C. psittaci* identified	NR	Blomqvist et al., 2012 [76]
Feral pigeons (*Columbia livia domestica*), ring-necked parakeets (*Psittacula krameri*), crows (*Corvus splendens*)	Psittacidae, Columbidae, Corvidae	India	Trapping	85 (55 pigeons, 19 parakeets, 11 crows)	Isolation and inoculation (faecal swabs/intestinal and visceral organs) Serology (indirect micro-immunofluorescense test (IMIFT) and ELISA)	*C. psittaci* (methods not spp.-specific)	• 26.3% prev. in ring-necked parakeets • 16.4% prev. in pigeons • 18.2% prev. in crows	NR	Chahota et al., 1997 [61]
Seabirds; 22 species *	Laridae, Alcidae	Bering Sea	NR	722	PCR (faeces) *ompA*, mpB, and 16S sequencing	*C. psittaci* *Chlamydiales*	• 0.1% *C. psittaci* prev. (*n* = 1 black-headed gull (*Larus ridibundus*))	NR	Christerson et al., 2010 [57]
Blue-fronted Amazon parrot (*Amazona aestiva*) and hyacinth macaw (*Anodorhynchus hyacinthinus*)	Psittacidae	Brazil	Nestlings (breeding monitoring)	77 (32 Amazon parrots nestlings, 45 macaw nestlings)	PCR (tracheal and cloacal swabs) Serology (CFT)	*C. psittaci* (methods not spp.-specific)	• 6.3% prevalence in Amazon parrots (cloacal swabs) • 26.7% prevalence in hyacinth macaws (cloacal swabs) 8.9% (tracheal swabs), 4.8% (CFT)	N	de Freitas Raso et al., 2006 [62]
Feral pigeons	Columbidae	Brazil	Live trapping	238	PCR (cloacal and tracheal swabs)	*C. psittaci*	• 16.8% *C. psittaci* prev. • Prev. ranged from 6.1% to 37.8% according to location	NR	de Lima et al., 2010 [69]
Blue-fronted Amazon parrot	Psittacidae	Bolivia	Live trapping	34	Serology (CFT)	*C. psittaci* (method not species-specific)	• 0% prevalence	N/A	Deem et al. 2005 [84]
Canada geese (*Branta* *canadensis*)	Anatidae	Belgium	Culling program	81	Serology (rMOMP-based ELISA) Inoculation and culture (pharyngeal swabs)	*C. psittaci*	• 93.6% seropositive • 58% of swabs were culture positive, but low culture score (low no. of viable organisms)	N	Dickx et al., 2013 [73]
Rosy-faced lovebirds (*Agapornis roseicollis);* 15 other species, including Passeriformes and Columbiformes *	Psittaculidae, Columbidae, Icteridae, Troglodytidae (…) *	USA	Live trapping	188 (46 lovebirds; 142 birds of other species)	PCR (conjunctival/ choanal and cloacal swabs) Serology (Elementary body agglutination (EBA))	*C. psittaci*	• 93% *C. psittaci* prev. and 76% seroprevalence in lovebirds • 10% *C. psittaci* prev. and 7% seroprevalence (in all other bird species combined)	NR	Dusek et al., 2018 [88]
Feral pigeons	Columbidae	Brazil	Live trapping	240	PCR (cloacal swabs)	*C. psittaci*	• 13% *C. psittaci* prevalence	NR	Ferreira et al., 2016 [68]
New Zealand bellbirds (*Anthornis melanura*; *n* = 4); rifleman (*Acathisitta* *chloris*; *n* = 3); hihi (*Notiomyces cincta*; *n* = 2); whitehead (*Mohoua albicilla*; *n* = 1)	Meliphagidae, Notiomystidae, Acanthisittidae, Mohouidae	New Zealand	Live trapping	10	PCR (cloacal swabs) Sequencing	*C. psittaci*	• 10% *C. psittaci* prevalence; one bird identified positive (a hihi) • First report of *C. psittaci* detection from a wild native bird in New Zealand	NR	Gartrell et al., 2012 [89]
Dusky-headed parakeets (*Aratinga* *weddellii*) and Tui parakeets (*Brotogeris sanctithomae*)	Psittacidae	Peru	Live trapping	48 (35 dusky-headed parakeets, 13 Tui parakeets)	Serology (CFT, Latex agglutination, EBA)	*C. psittaci* (methods not spp.-specific)	• 0% seroprevalence using any method	N/A	Gilardi et al., 1995 [85]
Fulmars (*Fulmarus glacialis*)	Procellaridae	Faroe Islands	Non-flying juveniles sampled	431 (juveniles)	PCR (cloacal swabs) *ompA* sequencing	*C. psittaci*	• 10% *C. psittaci* prevalence (range from 7% to 21% between locations) • 6BC strain identified in all positive samples	NR	Hermann et al., 2006 [74]
Great tits (*Parus major*; *n* = 318), blue tits (*Parus caerulus*; *n* = 43), marsh tits (*Parus palustris*; *n* = 32), coal tits (*Parus ater*; *n* = 3), willow tits (*Parus montanus*; *n* = 3)	Paridae	Germany	Live trapping (*n* = 389), necropsy (*n* = 6)	395	Inoculation and culture (cloacal and pharyngeal swabs) Organ tissues (necropsied birds)	*Chlamydia*	• 54.3% *Chlamydia* prevalence • Shedding varied according to swab site • Prevalence varied between host species; highest prevalence in blue tits, followed by great tits, then marsh tits • Repeated sampling of *n* = 38 individuals; 60.5% changed in *Chlamydia* status	N	Holzinger-Umlauf et al., 1997 [80]
Chinstrap penguins (*Pygoscelis antarcticus*) and Magellanic penguins (*Scheniscus magellanicus*); seabirds *	Sphenisicidae, Sterocorariidae, Laridae, Procellaridae, Chionidae	Antarctica, Chile	Live trapping (penguins), fresh faeces (seabirds)	527 (264 penguins; 263 seabirds)	PCR (cloacal swabs and faeces) Sequencing	*C. psittaci* *Chlamydiales*	• 18% *Chlamydiales* prevalence (Antarctica) • No *C. psittaci* detected in birds in Antarctica • 10% *C. psittaci* prevalence (Chile)	NR	Isaksson et al., 2015 [77]
43 species; 14 different orders *	Corvidae, Scolopacidae, Columbidae (…) *	South Korea	Rehabilitation centres	225	PCR (tracheal swabs and tissues) *ompA* sequencing	*C. psittaci* *Chlamydiales*	• 1.8% *C. psittaci* prev. (rook (*Corvus frugilegus*), Korean magpie (*Pica serica*), feral pigeon) • 0.9% *C. gallinacea* prev. (woodcock (*Scolopax rusticola*))	NR	Jeong et al., 2017 [36]
Raptors: osprey (*Pandion haliaetus*), great horned owl (*Bubo virginianus*), red-tailed hawk (*Buteo jamaicaensis*) (others not listed)	Accipitridae, Pandionidae, Strigidae (others not listed)	USA	Rehabilitation centres	82	PCR (oral and cloacal swabs) Sequencing	*C. psittaci* *Chlamydiales*	• 1 osprey was *C. psittaci*-positive • 1 red-tailed hawk was *Ca*, Rhabdochlamydia spp. positive	NR	Jouffroy et al., 2016 [75]
35 species; 15 orders *	Anatidae, Accipitridae, Passeridae (…) *	Poland	Hunting, culling programs, fishing bycatch, wildlife rehabilitation centres, community submissions	369	PCR (combined tissues and conjunctival swabs) Sequencing	*Chlamydia* (all species)	• 7.3% *Chlamydia* prevalence • *C. psittaci* and *C. trachomatis* identified • *Chlamydia* positive birds identified across eight orders	N	Krawiec et al., 2015 [52]
Hawks; *Buteo* genus	Accipitridae	USA	Live trapping	297	PCR (conjunctival, choanal, and cloacal swab) Sequencing Serology (EBA) and immunofluorescent antibody (IFA))	*Chlamydiaceae*	• 1.4% *Chlamydia* prev. (2 positive red-tailed hawks, 2 positive Swainson’s hawks (*Buteo swainsoni*)) • 0% seroprevalence	N	Luján-Vega et al., 2018 [37]
Feral pigeons; house sparrows	Columbidae	Iran	NR	150 (75 pigeons; 75 house sparrows)	PCR (cloacal swabs) *ompA* sequencing	*C. psittaci*	• 25.3% *C. psittaci* prev. in pigeons • 18.6% *C. psittaci* prev. in house sparrows • Genotypes A and B identified	NR	Mahzounieh et al., 2020 [90]
Feral pigeons, Eurasian collared doves (*Streptopelia decaocto*), wood pigeon (*Columba palumbus*)	Columbidae	Switzerland	Pigeon lofts, rehabilitation centres, culling programs	431	PCR (choanal/cloacal swabs and liver samples) DNA microarray assay 16S sequencing MLST	*C. psittaci* *Chlamydiaceae*	• 14.1% *Chlamydiaceae* prev. (feral pigeons) • 5.1% *Chlamydiaceae* prev. (collard dove) • 5.7% *Chlamydiacaeae* prev. (wood pigeon) • Prevalence in feral pigeons varied by location • 57.6% positive samples were *C. psittaci*, 5.4% of positive samples were *C. avium*	NR	Mattman et al., 2019 [47]
7 species, order Psittaciformes, Anseriformes, Passeriformes *	Cacatuidae, Anatidae, Rallidae, Artamidae	Australia	NR	124	PCR (conjunctival, choanal, and cloacal swabs) Cell culture	*C. psittaci*	• 0% prevalence; no wild birds tested positive	N/A	McElnea and Cross, 1999 [91]
Galapagos doves (*Zenaida* *galapagoensis*) and feral pigeons	Columbidae	The Galapagos Islands, Ecuador	Live trapping	133 (105 Galapagos doves, 28 feral pigeons)	PCR (cloacal swabs)	C. psittaci	• 6% *C. psittaci* prev. (Galapagos doves) • 0% *C. psittaci* prev. (feral pigeons) • Geographic variation in prev. (all positive cases occurred on one island)	NR	Padilla et al., 2004 [92]
Ring-necked parakeet	Psittacidae	France	Live trapping	85	PCR (cloacal swabs)	*C. psittaci* *C. avium* *Chlamydiaceae*	• 7.1% *Chlamydiaceae* prevalence • The chlamydial species was only identified to species level in one individual (*C. avium*)	NR	Pisanu et al. 2018 [49]
Red-tailed Amazon parrot (*Amazona brasiliensis*)	Psittacidae	Rasa Island, Brazil	Nestlings (breeding monitoring)	117 (nestlings)	PCR (tracheal and cloacal swabs)	*C. psittaci* (method not spp. specific)	• 1.2% prevalence (one positive sample identified)	N	Ribas et al. 2014 [82]
Feral pigeons	Columbidae	Germany	Management project	570	PCR (cloacal swabs and faeces) DNA microarray *ompA* sequencing	*C. psittaci* *Chlamydiaceae*	• 14.6% *Chlamydiaceae* prev. (swabs) and 10.4% *C. psittaci* prev. (swabs) • Faecal prev. higher than swabs • Temporal variation in *Chlamydiaceae* prev.; 9.3% prev. in 2009, compared to 19.3% in 2010 • *C. pecorum*, *C. abortus*, *C. trachomatis*, and unclassified *Chlamydiaceae* also identified	NR	Sachse et al., 2012 [66]
Feral pigeons	Columbidae	Thailand	NR	407	PCR (tracheal and cloacal swabs), *ompA* sequencing	*C. psittaci*	• 10.8% *C. psittaci* prevalence • Genotype B identified	N	Sariya et al., 2015 [71]
Raptors; 15 species *	Accipitridae, Falconidae, Tytonidae, Strigidae, Pandionidae	Germany	Veterinary submissions	39	PCR (lung and spleen)	*C. psittaci*	• 74% *C. psittaci* prevalence • No association of infection with sex, age, or year	NR	Schettler et al., 2003 [93]
Raptors; 346 diurnal birds of prey; 55 owls) *	Accipitridae, Pandionidae, Strigidae, Tytonidae, Falconidae	Germany	Rehabilitation centres	428	Serology (ELISA)	*C. psittaci*	• 63% seropositivity • Age association with seroprevalence; older birds more likely to test seropositive	NR	Schettler et al., 2001 [94]
10 species * majority of birds tested were Columbiformes	Columbidae, Turdidae, Anatidae (…)	U.K.	Rehabilitation centre	43	PCR (cloacal swabs)	*C. psittaci*	• 11.6% *C. psittaci* prevalence • All positive birds were Columbiformes • Positive pigeons were emaciated and anorexic, but no signs of respiratory distress	Y	Sharples and Baines, 2009 [95]
42 species *	Columbidae, Passeridae, Fringillidae (…) *	Switzerland	Rehabilitation centre	339	PCR (choanal and cloacal swabs, faecal swabs) *ompA* sequencing	*C. psittaci* *Chlamydiaceae*	• 0.9% *Chlamydiaceae* prev. (all Columbidae) • No other birds tested positive	NR	Stalder et al., 2020 [31]
Raptors (16 species); corvids (six species) *	Accipitridae, Falconidae, Strigidae, Tytonidae, Corvidae	Switzerland	Rehabilitation centres, community submissions, culling programs	594 (341 raptors, 253 corvids)	PCR (choanal and cloacal swabs, faecal swabs) *ompA* and 16S sequencing	*C. psittaci* *C. buteonis* *Chlamydiaceae*	• 23.7% *Chlamydiaceae* prev. (corvids) • 5.9% *Chlamydiaceae* prev. (raptors) • 0% *C. buteonis* prev.	N	Stalder et al., 2020 [34]
Feral pigeons	Columbidae	Poland	NR	101	PCR (cloacal and pharyngeal swabs) *ompA* sequencing	*C. psittaci*	• 3.9% *C. psittaci* prevalence • More pigeons were co-infected with *C. psittaci* and pigeon circovirus than with *C. psittaci* alone	N	Stenzel et al., 2014 [96]
Crimson rosella	Psittacidae	Australia	Live trapping	136	PCR (cloacal swabs) Serology (ELISA) 16S sequencing	*C. psittaci* *C. gallinacea* *Chlamydiales*	• 27.7% *Chlamydiales* prevalence • 6.2% *C. psittaci* prev. and 4.6% *C.* *gallinacea* prev. • 16% seroprevalence • Higher *Chlamydiales* prev. in non-breeding birds and female birds • Seroprevalence was highest in autumn and in non-breeding birds	N	Stokes et al., 2020 [46]
7 species; order Psittaciformes *	Psittacidae, Cacatuidae	Australia	Live trapping	132	PCR (cloacal swabs) Serology (ELISA) 16S and *ompA* sequencing	*C. psittaci* *C. gallinacea Chlamydiales*	• Overall *Chlamydiales* prevalence was 39.8% • *C. psittaci* prevalence was 9.8%, and *C. gallinacea* prevalence was 0.8% • Seroprevalence was 37.0% • Prevalence varied between species and location	N	Stokes et al., 2020b [33]
Long-billed corella (*Cacatua* *tenuirostris*), little corella (*Cacatua sanguinea*), sulfur-crested cockatoo (*Cacatua galerita*) and galah (*Eolophus roseicapillus*)	Cacatuidae	Australia	Live trapping and rehabilitation centres	55	PCR (choanal/cloacal swabs) Next-generation sequencing (NGS)	*C. psittaci*	• None PCR positive, but NGS identified *C. psittaci* in one little corella; hence, overall prevalence was 1.8%	Y	Sutherland et al. 2019 [63]
33 species; 16 families *	Accipitridae, Anatidae, Corvidae (…) *	Poland	Rehabilitation centres; some free-living birds captured	894	PCR (cloacal or faecal swabs) *ompA* and rrn sequencing	*C. psittaci* *C. abortus* *Chlamydiaceae*	• 14.8% *Chlamydiaceae* prev. (all birds tested) • 19.7% *Chlamydiaceae* prev. (Anatidae) • 13.4% *Chlamydiaceae* prev. (Corvidae) • *C. psittaci/C. abortus* intermediate isolates identified	NR	Szymańska-Czerwińska et al., 2017 [29]
Common swift (*Apus apus*)	Apodidae	Germany	Community veterinary submissions	243	PCR (pooled organs)	*C. psittaci* *Chlamydia*	• 0% prev. (no birds tested positive over 9 years)	N/A	Tiyawattanaroj et al. 2021 [86]
Red-tailed Amazon parrot	Psittacidae	Rasa Island, Brazil	Nestlings (breeding monitoring)	74 (nestlings)	PCR (cloacal and oropharyngeal swabs) Serology (ELISA)	*C. psittaci* (method not spp. specific)	• 0% prevalence	N/A	Vaz et al. 2017 [83]
African sacred ibis (*Threskiornis aethiopicus*)	Threskiornithidae	France	Culling program	70	PCR (cloacal swabs) Culture and inoculation	*C. psittaci* *Chlamydiaceae*	• 11% *Chlamydiaceae* prev. • 1.4% *C. psittaci* prev. and • 4.3% *Ca*. C. ibidis prev. • *Ca*. C. ibidis identified and proposed as a new species	N	Vorimore et al., 2013 [50]
Greater flamingo (*Phoenicopterus roseus*)	Phoenicopteridae	France	Live trapping	404	PCR (cloacal swabs) Isolation and cell culture Sequencing	*C. psittaci* *C. avium* *C. gallinacean* *Ca.* C. ibidis	• 30.9% (125/404) chlamydial positive, but not for known species • Three isolates were cultured, with two new species identified and proposed, in a new genus, *Chlamydiifrater* gen. nov.	N	Vorimore et al., 2021 [78]
Feral pigeons	Columbidae	Thailand	Live trapping (public locations)	150	PCR (cloacal swabs) Isolation and inoculation	*C. psittaci*	• 1.3% *C. psittaci* prevalence	N	Wannaratana et al., 2017 [70]
Songbirds (*n* = 527; 11 families) Pigeons (*n* = 84; Columbidae) Waterfowl (*n* = 442; 5 families) *	Columbidae, Fringillidae, Turdidae (…) *	Switzerland	Collected through avian influenza surveillance, live trapping (pigeons), and hunters (cormorants)	1091	PCR (cloacal swabs) 16S Sequencing	*C. psittaci* *Chlamydiaceae*	• 3.3% *C. psittaci* prev. in feral pigeons • 0.4% *Chlamydiaceae* prev. in songbirds (Passeriformes) • 4% *Chlamydiaceae* prev. in waterfowl (all were tufted ducks (*Aythya fuligula*) and pochards (*Aythya farina*))	NR	Zweifel et al., 2009 [30]

* For the list of all species and families tested, refer to the publication. ** Y indicates Yes; N indicates No; NR indicates Not Recorded.

## Data Availability

All publications or data cited in this review are already in the public domain.
